# Evaluating Helicobacter pylori Infection as a Risk Factor for Iron Deficiency Anemia: A Case-Control Approach

**DOI:** 10.7759/cureus.84340

**Published:** 2025-05-18

**Authors:** Dipendra Singh, Varun Upadhyay, Sunny Bhushan Singh, David Rimal, Objan K Rawal, Roshani Lamichhane

**Affiliations:** 1 Orthopedics, Soo-Jung Hospital Doti, Rajpur Doti, NPL; 2 Internal Medicine, Yeti Hospital, Kathmandu, NPL; 3 Internal Medicine, B.P. Koirala Institute of Health Sciences, Dharan, NPL

**Keywords:** gastrointestinal bleeding, helicobacter pylori, hemoglobin levels, iron deficiency anemia (ida), serum ferritin, serum iron

## Abstract

Background

The medical community identifies *Helicobacter pylori *(*H. pylori*) infection as a growing cause of extra-gastrointestinal manifestations, which primarily affect iron deficiency anemia (IDA). The purpose of this research was to analyze how *H. pylori* infection relates to IDA through the case-control method.

Method

This study enrolled 48 individuals, divided into 24 (50%) cases of diagnosed IDA patients and 24 (50%) healthy controls who were matched by age and sex at Soo-Jung Hospital Doti, Rajpur Doti, Nepal. The study enrolled all subjects for laboratory examinations of blood tests, along with *H. pylori* infection assessments via stool antigen testing or urea breath tests. The researchers documented dietary patterns, non-steroidal anti-inflammatory drug (NSAID) use, gastrointestinal symptoms, and bleeding history. The investigators analyzed the data through SPSS Version 22 (IBM Corp., Armonk, NY) and conducted independent t-tests and chi-square tests to compare groups, with statistical significance set at p < 0.05.

Results

Research findings indicated that subjects with IDA presented a higher rate of *H. pylori* positivity, 21 (87.5%), compared to the control group, 7 (29.2%) (p < 0.001). The mean levels of hemoglobin, ferritin, and serum iron were significantly reduced in cases (9.7 g/dL versus 13.0 g/dL) (17.2 ng/mL versus 58.1 ng/mL), while they showed elevated total iron-binding capacity and decreased mean cellular volume levels. The proportion of patients with poor nutritional status and NSAID use was higher among participants who tested positive for *H. pylori*.

Conclusion

The evidence indicated that *H. pylori *infection establishes a direct link to the development of IDA. The combined assessment and therapy of *H. pylori* infection in anemic patients demonstrated promise for proper management and anemia resolution, particularly when patients have an inadequate diet or use NSAIDs.

## Introduction

Iron deficiency is the most common worldwide micronutrient deficiency, affecting almost one in six people or approximately 16.7% of the population. This issue causes 423.7 disability-adjusted life-years (DALYs) per 100,000 people [1]. Insufficient hemoglobin synthesis occurs because of insufficient available iron, which causes iron deficiency anemia (IDA) symptoms, including fatigue, pallor, and reduced cognitive and physical abilities [2]. The main sources of IDA involve poor nutrition and persistent blood loss, yet researchers now show evidence that *Helicobacter pylori* (*H. pylori*), in particular, has started to affect iron metabolism. The Gram-negative pathogen *H. pylori* establish itself in the gastric mucosal tissues and contributes to all major inflammatory diseases of the digestive system, including gastritis, peptic ulcers, and gastric malignancies [3].

The medical effects of this bacterial infection extend beyond the destruction of the gastrointestinal (GI) tract. Several studies demonstrated that *H. pylori* creates IDA by causing chronic gastric inflammation, which results in microvascular bleeding and decreased gastric iron absorption due to pH changes and bacterial iron use [4]. These mechanisms pose a serious concern for populations with restricted dietary access to iron or those who use non-steroidal anti-inflammatory drugs (NSAIDs), as these factors independently damage mucosal surfaces [5]. The relationship between *H. pylori* infection and IDA remains unexplored in various territories, particularly throughout South Asia [6]. Research studies examining the impact of dietary habits, together with NSAID intake, on *H. pylori*-related anemia remain scarce in local regions [7].

Researchers performed a case-control study to determine how *H. pylori* infection relates to IDA, while also analyzing dietary choices, stomach discomforts, and NSAID exposure. Understanding this relationship helps health practitioners to identify treatable anemia causes through strategic interventions, particularly within resource-constrained environments, which face significant public health challenges due to reduced quality of life associated with anemia.

## Materials and methods

The researchers performed this case-control evaluation to determine whether *H. pylori* infection is associated with IDA. The study was conducted over three months from January 2025 to March 2025 and included 48 participants who visited a clinical diagnostic center at Soo-Jung Hospital Doti, Rajpur Doti, Nepal (Ref:29/12/2024). It included 24 (50%) patients diagnosed with IDA (cases) and 24 (50%) voluntarily enrolled healthy subjects (controls), who were matched by age and gender. All participants filled out a standardized consent form for participation in an experiment. The sample size was calculated using OpenEpi software version 3.0.0. The calculation was based on the expected *H. pylori* infection among IDA patients. A case-control ratio of 1:1 was used with a confidence interval of 95%, which is canonical.

To qualify as a case, participants needed to exhibit low hemoglobin levels along with decreased ferritin levels, but those with recent iron supplementation, chronic kidney disease, or active malignancy were excluded from the study. A stool antigen test and urea breath test served as the *H. pylori* screening methods for all participants. The laboratory analysis involved measuring hemoglobin, mean cellular volume (MCV), serum ferritin, serum iron, and total iron-binding capacity (TIBC). A structured questionnaire was used to collect information on dietary habits of patients, NSAID use, GI symptoms, and signs of bleeding. SPSS Version 22.0 (IBM Corp., Armonk, NY) was used to process the dataset. The statistical analysis employed descriptive methods, producing results including frequencies, percentages, means, and standard deviations. Statistical tests included independent t-tests for continuous variables and chi-square tests for categorical data to compare the two groups. A p-value of less than 0.05 was considered statistically significant.

## Results

This research study demonstrated a strong association between *H. pylori* infection and IDA, as shown in Table 1. A comprehensive comparison of IDA patients and healthy participants yielded consistent results in both blood analyses and clinical measurements. The study data demonstrated statistically significant differences in infection rates, blood values, dietary patterns, GI discomfort, and NSAID use.

**Table 1:** Distribution of H. pylori status among cases and controls

Case/Control	*H. pylori* Positive, n (%)	*H. pylori,* Negative n (%)	Total
Case	21 (87.5%)	3 (12.5%)	24
Control	7 (29.2%)	17 (70.8%)	24
Total	28 (58.3%)	20 (41.7%)	48

Research results demonstrated that *H. pylori* infection occurred predominantly in patients with IDA, with a prevalence of 21 (87.5%) compared to 7 (29.2%) in the control group. The data revealed that *H. pylori* testing provides strong evidence of the bacterium’s association with IDA development. Table 2 highlights the comparison of hematological parameters among cases and controls.

**Table 2:** Comparison of hematological parameters between IDA cases and healthy controls Continuous variables are summarized as mean ± SD; independent two‐sample t‐test statistics (t-value) and p-values. Reference ranges: hemoglobin (male: 13.8–17.2 g/dL, female: 12.1–15.1 g/dL); ferritin (male: 24–336 ng/mL, female: 11–307 ng/mL); serum iron (male: 65–176 µg/dL, female: 50–170 µg/dL); TIBC: 250–450 µg/dL; MCV: 80–100 fL. IDA, iron deficiency anemia; TIBC, total iron-binding capacity; MCV, mean cellular volume

Parameter	Cases (Mean ± SD)	Controls (Mean ± SD)	t-value	p-value
Hemoglobin (g/dL)	9.74 ± 1.00	13.00 ± 1.01	–11.20	< 0.001
Serum ferritin (ng/mL)	17.21 ± 6.44	58.13 ± 14.27	–13.68	< 0.001
Serum iron (µg/dL)	38.33 ± 13.47	89.17 ± 18.95	–11.66	< 0.001
TIBC (µg/dL)	412.04 ± 32.67	323.54 ± 34.64	9.97	< 0.001
MCV (fL)	70.38 ± 5.43	84.42 ± 4.12	–10.51	< 0.001

Continuous hematological parameters were compared between IDA cases and controls using independent two-sample t-tests. All five variables differed highly significantly between groups (hemoglobin: t = -11.20, p < 0.001; serum ferritin: t = -13.68, p < 0.001; serum iron: t = -11.66, p < 0.001; TIBC: t = 9.97, p < 0.001; MCV: t = -10.51, p < 0.001). Categorical variables (GI symptoms, GI bleeding, poor nutritional status, and NSAID use) were compared between *H. pylori*-positive and *H. pylori*-negative participants using chi-square tests. The results are shown in Table 3.

**Table 3:** Chi‐square analysis of categorical outcomes by H. pylori status Rows list number (%) in each group; χ², df = 1, and exact p-values from Pearson’s chi-square test. Categorical variables were analyzed using Pearson’s chi-square test; χ² = chi-square statistic, df = degrees of freedom (1), p-value = two-sided significance. A p-value < 0.05 was considered statistically significant. GI, gastrointestinal; NSAID, non-steroidal anti-inflammatory drug

Outcome	*H. pylori *Positive (n=28)	*H. pylori *Negative (n=20)	χ²	df	p-value
GI symptoms	25 (89.3%)	8 (40.0%)	10.996	1	0.0009
GI bleeding	12 (42.9%)	2 (10.0%)	4.610	1	0.0318
Poor nutritional status	23 (82.1%)	6 (30.0%)	11.173	1	0.0008
NSAID use	18 (64.3%)	4 (20.0%)	7.519	1	0.0061

Patients infected with *H. pylori* experienced more GI symptoms, 25 (89.3%), combined with GI bleeding in 12 (42.9%). These symptoms were less frequent in *H. pylori*-negative individuals, eight (40.0%) and two (10.0%) cases, respectively. These data show that *H. pylori* is involved in causing GI problems, which leads to iron deficiency. Poor dietary intake affected 23 (82.1%) of *H. pylori*-positive patients compared to only six (30%) of *H. pylori*-negative individuals. The nutritional deficiencies in infected patients create conditions that enhance the negative impact on iron absorption. The frequency of NSAID use was 18 (64.3%) among individuals infected with *H. pylori *compared to only four (20%) among those without the infection. The effect of NSAIDs and *H. pylori* increases the risk of GI bleeding and iron loss.

Overall, these findings confirmed that there was a strong association between *H. pylori *infection and both marked abnormalities in iron‐related hematological parameters and higher rates of GI symptoms, bleeding, poor nutritional status, and NSAID use among IDA patients.

## Discussion

Scientists studied the relationship between *H. pylori* infection and IDA through a case-control investigation, which included testing hematological data, gastric symptoms, NSAID intake, and dietary analysis. Research results supported the role of *H. pylori* infection in IDA by showing that the infection leads to alterations in iron parameters, including hemoglobin, serum ferritin, serum iron, TIBC, and MCV [8]. IDA-affected participants showed significantly lower average blood hemoglobin levels at 9.74 ± 1.00 g/dL, along with decreased serum ferritin and serum iron levels, while their TIBC values remained elevated. These changes support the established pathophysiological processes of *H. pylori*-related iron regulation dysregulation [9]. The persistent gastric inflammation caused by *H. pylori* blocks iron absorption, thereby triggering occasional bleeding inside the digestive tract. The bacterial strains compete with the human body for iron access, thus causing a condition referred to as functional iron depletion [10].

The study revealed higher percentages of *H. pylori *infections in the IDA patient group compared to the control group, indicating that the relationship between this bacterium and IDA could be causal [11]. The observational data supported international studies by linking *H. pylori* to refractory and unexplained IDA, especially when patients had restricted iron diets or took NSAIDs. The rates of dietary insufficiency and NSAID use were higher in patients with IDA [12]. People who maintain a diet lacking iron-rich foods cannot restore their iron levels, and the use of NSAIDs exacerbates mucosal damage, thereby increasing the risk of bleeding [13]. The combined impact of *H. pylori* infection and other environmental factors likely worsened iron deficiency among the participants [14].

Several drawbacks existed in the study, which included the small sample size, the solitary hospital location, and the absence of endoscopic gastric examinations. This research contributed critical knowledge about the various contributing factors of IDA despite missing confirmation of GI infections from endoscopic tests in clinical settings [15]. Testing for *H. pylori *infection should be performed for individuals with IDA, when dietary challenges and NSAID consumption are a part of their medical history. Additions of early treatment methods for *H. pylori* combined with non-invasive diagnostic technologies will help enhance care for affected individuals [16].

A limitation of this study stemmed from its small sample size and the inclusion of only a single health center. A test for gastric lesions using endoscopy was omitted from the study evaluation. Participants reported their dietary consumption and NSAID use throughout the study, which might have led to biased recall data. The observational nature prevents the authors from establishing causal connections between the studied variables.

## Conclusions

The research established a direct link between *H. pylori* infection and IDA, demonstrating significant differences between patients with and without the infection. The management of *H. pylori *infection became more complicated when patients had insufficient nutrition and consumed NSAIDs, as both factors contributed to ongoing iron depletion. Healthcare providers should diagnose *H. pylori* infection in patients with anemia, as successful treatment could help restore iron levels and reduce disease symptoms, particularly when patients use NSAIDs or have inadequate diets. The findings suggest that primary healthcare facilities and tertiary care settings should implement comprehensive screening programs for unexplained anemia, as such conditions are commonly encountered in clinical practice.
